# Therapeutic Response-Based Reclassification of Multiple Tumor Subtypes Reveals Intrinsic Molecular Concordance of Therapy Across Histologically Disparate Cancers

**DOI:** 10.3389/fcell.2021.773101

**Published:** 2021-11-12

**Authors:** Yue Xu, Jie Zheng, Zhaoqing Cai, Wang Li, Jens Köhler, Yao Dai, Xiaojie Cheng, Tao Wu, Fan Zhang, Haiyun Wang

**Affiliations:** ^1^ School of Life Sciences and Technology, Tongji University, Shanghai, China; ^2^ Department of Thoracic Surgery, Shanghai Pulmonary Hospital, Tongji University School of Medicine, Shanghai, China; ^3^ Department of Medical Oncology, Dana-Farber Cancer Institute, Boston, MA, United States

**Keywords:** tumor classification, OMICS data, pharmacological subtypes, precision medicine, CGP dataset

## Abstract

Cancers that are histologically defined as the same type of cancer often need a distinct therapy based on underlying heterogeneity; likewise, histologically disparate cancers can require similar treatment approaches due to intrinsic similarities. A comprehensive analysis integrated with drug response data and molecular alterations, particularly to reveal therapeutic concordance mechanisms across histologically disparate tumor subtypes, has not yet been fully exploited. In this study, we integrated pharmacological, genomic, and transcriptomic profiling data provided from the Cancer Genome Project (CGP) in a systematic in silico investigation of the pharmacological subtypes of cancers and the intrinsic concordance of molecular mechanisms leading to similar therapeutic responses across histologically disparate tumor subtypes. We further developed a novel approach to redefine cell-to-cell similarity and drug-to-drug similarity from the therapeutic concordance, providing a new point of view to study cancer heterogeneity. This study demonstrates how pharmacological and omics data can be used to systematically classify cancers in terms of response to various compounds and provides us with a purely therapy-oriented perspective to view tumor classifications independent of histology subtypes. The knowledge of pharmacological subtypes of 367 drugs are available via our website (http://www.hywanglab.cn/dtdb/), providing the resources for precision medicine in the perspective of therapeutic response-based re-classification of tumor.

## Introduction

Traditional tumor classification based on histopathologic diagnosis and the TNM staging system offers highly practical guidance for surgical resection, regional radiotherapy or chemotherapies. However, due to complex intertumor and intratumor heterogeneity, cancer patients with such histological diagnoses often suffer from receiving effective drug treatment and therapeutic resistance (Bedard et al., 2013; Arozarena and Wellbrock, 2019; Ding et al., 2019). One of the key mechanisms behind this significant challenge is the fact that cancers from the same tissue of origin often present quite different mechanisms of oncogenesis at the molecular level (Bedard et al., 2013; Arozarena and Wellbrock, 2019). Decades of studies have focused on finding molecular subtypes within histopathologically defined tumor types by analyzing large-scale genomic, transcriptomic, proteomic, and epigenomic alterations (Ko et al., 2020). As a result, a variety of molecular signatures have been identified to distinguish intrinsic molecular subtypes associated with patient survival, prognosis and response to different therapeutic modalities (Gay et al., 2021). For example, BRAF mutation in melanoma (Menzer et al., 2019); EGFR-mutant lung adenocarcinomas (Marcoux et al., 2019); luminal A, luminal B, HER2-enriched, basal-like and normal-like subtypes in breast cancer (Prat et al., 2012; Rueda et al., 2019; Waks and Winer, 2019); and four prominent genetic subtypes in Diffuse large B-cell lymphoma (DLBCL), termed MCD based on the co-occurrence of MYD88L265P and CD79B mutations, BN2 based on BCL6 fusions and NOTCH2 mutations, N1 based on NOTCH1 mutations, and EZB based on EZH2 mutations and BCL2 translocations are several well-established molecular subtypes with explicit clinical significance (Schmitz et al., 2018).

With these advances in challenging cancer heterogeneity by identifying subtypes within the same tissues, emerging studies are uncovering facts in the other direction that cancers across disparate tissues of origin can explicitly share common molecular mechanism of oncogenesis (Hoadley et al., 2014; Lu et al., 2019; Petralia et al., 2020). For example, one study based on a large-scale genomic analysis revealed that lung squamous, head and neck, and a subset of bladder cancers shared highly concordant signatures typified by TP53 alterations, TP63 amplifications, and high expression of immune and proliferation pathway genes, implicating that those different cancers perhaps require similar treatment approaches (Hoadley et al., 2014). Another study based on large-scale genomic data found that TP53 and KRAS were mutually exclusive in COAD, READ, and LUAD, but significantly coexisted in PAAD. These observations reveal the feasibility of considering the same treatment strategy in different tumor types (Drilon et al., 2018). Similarly, HER2-targeted therapy may be applied to other cancer types analogous to breast cancer because ERBB2/HER2, which can be amplified in breast cancer, is also mutated and/or amplified in subsets of glioblastoma and gastric, serous endometrial, bladder and lung cancers (Cancer Genome Atlas Research et al., 2013). In recent years, the FDA granted approval to larotrectinib, which had marked and durable antitumor activity in a variety of patients with RTK fusion-positive cancer, regardless of age or tumor tissue type (Drilon et al., 2018). Such examples explicitly illuminate a distinct avenue for reclassifying multiple tumor types independent of histopathologic diagnosis, highlighting that treatment approaches specifically discovered in one disease can be applied to another due to their intrinsic concordance of molecular patterns.

Currently, emerging efforts, such as pan-cancer analysis projects, are being conducted to comprehensively define commonalities and differences across cancer types and tissues of origin (Cancer Genome Atlas Research et al., 2013). Nonetheless, integrative analysis, particularly towards revealing intrinsic therapeutic concordance across histologically disparate tumor subtypes, has not yet been fully exploited. Currently, a large-scale pharmacogenomics study, the Cancer Genome Project (CGP), provides high-throughput genomic, expression information and pharmacological profiling of anticancer drugs across hundreds of cell lines that represent explicit molecular subtypes of histologically defined tumors (Garnett et al., 2012). Hence, we integrated drug response information to pharmacological reclassify tumor subtypes regardless of their tissues of origin. Tumor cells in the same class present similar drug responses, and those in different classes show varied drug responses. Furthermore, by integrating genomic alteration and expression information, we unravelled the intrinsic concordant molecular mechanism associated with the common drug response across histologically disparate cancers. Importantly, this research provides us with a purely therapy-oriented perspective to re-examine tumor classifications independent of histology subtypes.

## Materials and Methods

Data from a large-scale pharmacogenomics study, the Cancer Genome Project (CGP), was accessible from its website: http://www.cancerrxgene.org. Gene expression, mutation and drug sensitivity data were downloaded. The CGP dataset includes 987 cell lines, genome-wide analysis of mutations, copy number variations and expression profiling, as well as the presence of commonly rearranged cancer genes, and 367 pharmacological profiles (dataset version 2020) (Garnett et al., 2012). For pharmacological assays, the dose range of drugs varied greatly for the different drugs. Generally, about 96% drugs used the minimal tested concentration below 0.07820 uM and the maximal tested concentrated below 20 uM. In these data, the natural logarithm of the Half maximal inhibitory concentration (IC50) value represents the drug sensitivity value. IC50 is the half maximal inhibitory concentration of an anticancer drug, and a lower value means higher sensitivity. Cell lines cannot be classified according to histological subtypes provided by TCGA, labelled as “UNCLASSIFIED”, were excluded from our study.

### Generating the Pharmacological Subtypes Tree

The cell lines, IC50 of which are smaller than maximal tested concentrations, are defined as sensitive, otherwise resistant. Our method iteratively splits the cancer cells into two groups in a way that gains the best separation of drug sensitivity between two groups until reaching two terminal conditions: The *p* value of the difference of two groups’ drug sensitivity values is smaller than 0.05 or all cancer cells in a node are sensitive or resistant.

Suppose the set of all cells is $S_{\mathrm{total}}$ , and each cell has its drug sensitivity value. The procedure to grow a pharmacological subtype tree is as follows:

1) Define the root node. Set S as the set of cancer cells in this node, and assign $S_{\mathrm{total}}$ to S .
2) Define the root node as the current node.
3) N denotes the number of cells in the current node. If N is smaller than 6 or all cells in such node are sensitive or resistant, finish the procedure of growing the tree. Otherwise, a heuristic algorithm splits S into two child nodes, the left node and the right node, which achieves the greatest drug sensitivity difference.
4) If no way can be found to split the current node ( p < 0.05), finish the procedure of growing the tree. Otherwise, the current node is split into two child nodes.
5) Define the left node and right node. Suppose the set of cells in the left node is $S_{l}$ and the set in the right node is $S_{r}$ . $S_{l}$ and $S_{r}$ are the subsets of S .
6) Define the left node as the current node. Set S as the set of cells in this node, and assign $S_{l}$ to S . Repeat steps 3–5.
7) Define the right node as the current node. Set S as the set of cells in this node, and assign $S_{r}$ to S . Repeat steps 3–5.

### Algorithm for Splitting the Node

We used a heuristic algorithm to search for a reasonable number of divisions to split S into $S_{l}$ and $S_{r}$ . Suppose the number of the cells set is ns . We first sorted the cells by IC50 ascending and divided the cells with higher drug sensitivity into the $S_{l}$ group and the cells with lower drug sensitivity into the $S_{r}$ group. Since each node at least 6 cells, a total of ns−5 divisions were considered.

For each division, the Mann-Whitney *U* test was applied to calculate the p value of drug sensitivity between $S_{l}$ and $S_{r}$ . The division with the smallest p value was selected as the optimized one to split the cancer cells in the current node.

### The Cell Similarity and the Drug Similarity

The similarity of cells is calculated based on their response to the different drugs. For each drug k , a pharmacological subtypes tree $S^{k}$ is generated, consisting of n subtypes:

$$S^{k}=\left(S_{1}^{k},S_{2}^{k},\ldots ,\;S_{n}^{k}\right)={\left(S_{t}^{k}\right)}_{t=1\to n}$$

Based on this tree, a matrix that defines a similarity of cancer cells, regarding whether they are in the same subtype and their drug response to drug k , is calculated. We assume there are m cell lines tested for drug k . The cell lines are labelled as sensitive, resistant, or NA (not available). A sensitive cell line has its IC50 value smaller than maximum tested concentration; a resistant cell line has its IC50 value greater than maximal tested concentration. For two given cell lines i and j , their similarity matrix $\left(D_{ij}^{k}\right)$ is defined as:

$$D_{ij}^{k}=\{\begin{array}{c} 1,\;\mathrm{if}\left(i,j\right)\in S_{t}^{k}\;\mathrm{and}\;\mathrm{both}\;\mathrm{are}\;\mathrm{labelled}\;\mathrm{as}\;\mathrm{sensitive}\;\mathrm{ones};\\ -1,\;\mathrm{if}\left(i,j\right)\in S_{t}^{k}\;\mathrm{and}\;\mathrm{both}\;\mathrm{are}\;\mathrm{labelled}\;\mathrm{as}\;\mathrm{resistant}\;\mathrm{ones};\\ 0,\;\mathrm{otherwise}. \end{array}$$

If two cell lines i and j are in the same subtype and both are sensitive to drug k , their score is 1, and −1 for both cell lines with a resistant response, while if they are not in the same subtype or show opposite response to the drug, their score is 0. A m×m similarity matrix of cell lines is then generated.

Then, the similarity of cell lines i and j can be further calculated as:

$${\mathrm{Cell}}_{\mathrm{sim}}=\frac{\sum_{k=1}^{q}\left|D_{ij}^{k}\right|}{q},\;i\neq j$$

Wherein i and j denote the cell lines tested for q drugs.

Furthermore, for two given drugs a and b , their similarity is calculated as the summarized similarity across the same cell lines which are tested for both drugs.

$$\mathrm{Dru}g_{\mathrm{sim}}=\frac{\sum_{i=1}^{m}\sum_{j=1}^{m}D_{ij}^{a}\cdot D_{ij}^{b}}{\sum_{i=1}^{m}\sum_{j=1}^{m}D_{ij}^{a}\cdot D_{ij}^{a}+\sum_{i=1}^{m}\sum_{j=1}^{m}D_{ij}^{b}\cdot D_{ij}^{b}-\sum_{i=1}^{m}\sum_{j=1}^{m}D_{ij}^{a}\cdot D_{ij}^{b}},\;i\neq j$$

Wherein i and j denote the cell lines tested for both drugs, and m is the total number of the same cell lines.

### Connecting Pharmacological Subtypes With Genomic Alterations

The maximum concentration of the drug was used to distinguish between the resistant pharmacological subtypes and sensitive pharmacological subtypes. The chi-squared test was used to calculate the connection between genomic alterations, including mutation and translocation, and pharmacological subtypes. Genomic alterations were determined with *p* values corrected with the Benjamini-Hochberg method for controlling the false discovery rate (Benjamini and Hochberg, 1995). Here we used the corrected *p* value <0.1 as the threshold. We defined two types of connections: sensitive and resistant. If pharmacological subtypes with higher drug sensitivity had more frequent alteration occurrences, we defined this connection as sensitive. If the opposite was true, we defined the connection as resistant.

The connection between mRNA expression and pharmacological subtypes was determined with *p* values from the student *t* test corrected with the Benjamini-Hochberg method. If pharmacological subtypes with higher drug sensitivity had higher expression, we defined this connection as sensitive. If the opposite was true, we defined the connection as resistant. Here we used the corrected *p* value <0.1 as the threshold. To obtain more confident connections, the Pearson correlation coefficient (PCC) was used to measure the correlation between drug sensitivity (IC50) and gene expression in the sensitive subtypes, and only genes with PCC greater than 0.3 were considered. The individual subtypes with sensitive and resistant cells mixed were excluded in the above analyses.

### Gene Set Enrichment Analysis

Gene set enrichment analysis (GSEA) (Subramanian et al., 2005) was employed to determine the 333 gene sets from KEGG, enriched by a pre-ranked list of all genes, which were sorted by the statistical significance of differential expression defined by DESeq2 analysis (Love et al., 2014). Gene sets with FDR <0.05 were statistically significant.

### Statistical Analysis

The Fisher’s exact test was used to respectively determine whether there is a significant association between pathways and clusters of drugs determined by the number of drug pharmacological subtypes across 6 cancer types or not, whether any difference of phenotypes (cancers/pathways) in clusters of cell lines/drugs derived from the similarity matrix was significant, and whether cancer censor genes are enriched in a list of genes with the most connections to pharmacological subtypes. A hypothesis test based on hypergeometric distribution was used to determine whether a histological cancer is enriched in the most sensitive pharmacological subtype of a drug. The *p* value <0.05 was regarded as statistically significant. Kolmogorov-Smirnov test (K-S test) was employed to compare cumulative distribution function (CDF) of the number of pharmacological subtypes across the drugs between two histological cancers.

## Results

We analysed 367 drugs in the CGP dataset and established their pharmacological subtype trees. The leaf node in each tree represents a pharmacological subtype of cancers, which is composed of cells from the histological disparate tumor subtypes. We further employed the mRNA expression profile and mutation/fusion profile to connect the molecular alterations with pharmacological subtypes, delineating a novel perspective to re-classify the tumor according to therapeutic response dependent of the intrinsic concordant molecular mechanism, by regardless of tissues of origin. The cell similarity and the drug similarity were also redefined based on the pharmacological subtypes.

### Pharmacological Subtypes of Cancers

**FIGURE 1:**
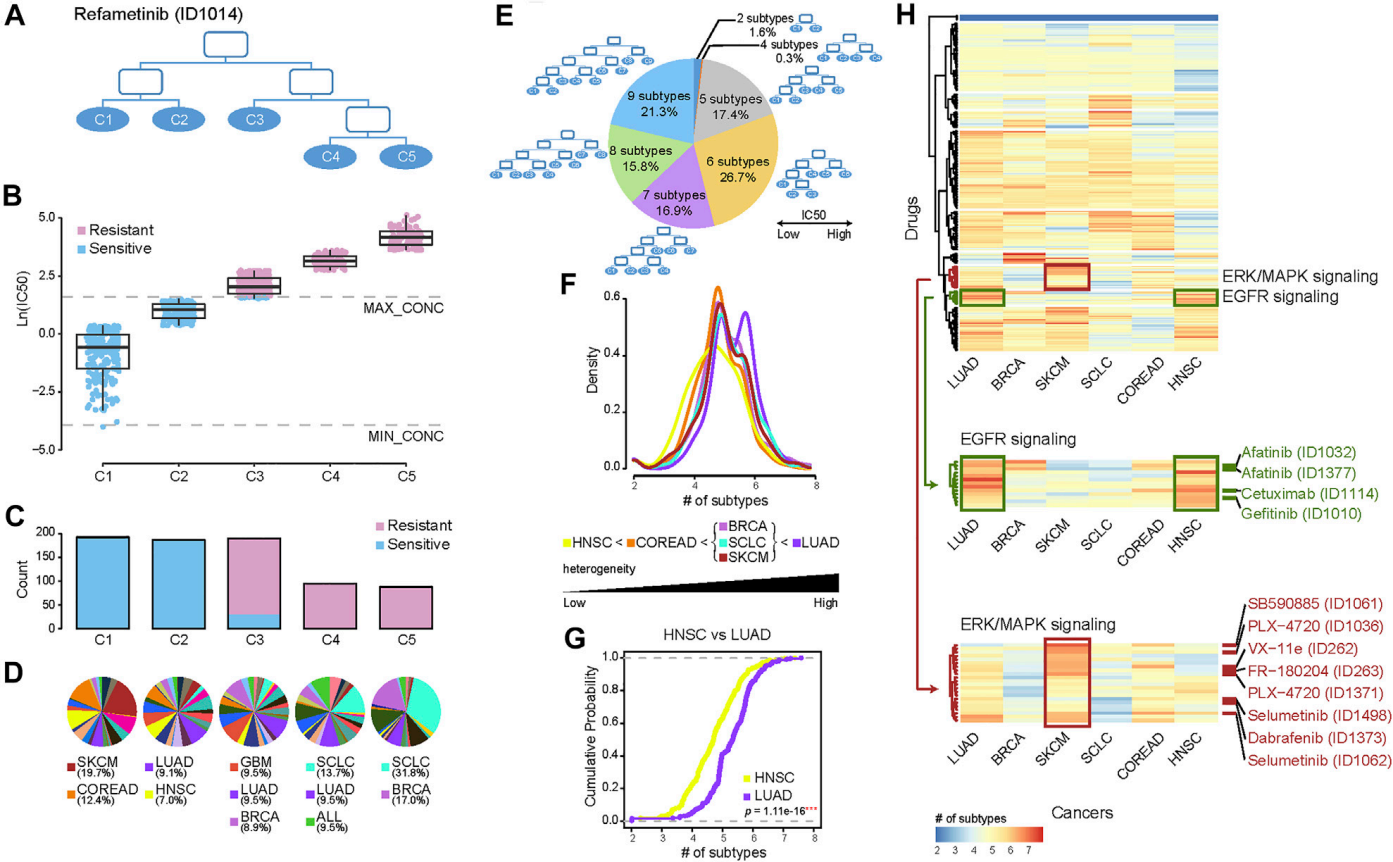
Re-classifying cancers to generate pharmacological subtypes and drug sensitivity profiles. **(A)** Pharmacological subtype tree of the drug Refametinib. Cancer cells were classified into five pharmacological subtypes based on their drug sensitivity to MEK inhibitor Refametinib. **(B)** The boxplot shows the drug sensitivities of five subtypes (leaf nodes): C1, C2, C3, C4, and C5, indicating a gradually increasing degree of resistance to the drug Refametinib. Blue points refer to cell lines sensitive to the drug, while red points resistant. **(C)** The barplot shows the number of cell lines in each subtype. **(D)** The pie chart shows the distribution of histological subtypes across pharmacological subtypes. **(E)** The pie chart illustrates the numerical proportion of different degrees of the furthest divisible pharmacological subtypes. Cancer cells could be maximally classified into up to 9 subtypes for different drugs. Among them, 7–9 subtypes accounted for more than half of drugs. **(F)** The density of the number of pharmacological subtypes indicates the varied treatment heterogeneity across six histological cancers, in which the average number of cell lines among all drugs are over 35. **(G)** The empirical cumulative distribution function visualizes the density of drug subtypes in LUAD vs. HNSC. *p* value was calculated by using the Kolmogorov-Smirnov test (K-S test). **(H)** Hierarchical clustering of the number of pharmacological subtypes across six cancers. Each row in the heatmap represents a drug, and each cell in the heatmap represents the number of subtypes of the cancer in the drug. The red color indicates more subtypes, and the blue color less subtypes. There are more pharmacological subtypes of EGFR inhibitors (green drugs) in LUAD and HNSC, and more subtypes of inhibitors targeting in ERK/MAPK signalling (red drugs) in SKCM.

We built a pharmacological tree for each drug based on the divisibility of the drug sensitivity of cancer cells. Taking the tree of the MEK1/2 inhibitor Refametinib as an example (Figure 1A), all cells in the root node were first divided into left and right child nodes with relatively high and low sensitivity, respectively, and further cells in these two nodes were capable of being divided into five final subgroups, C1, C2, C3, C4, and C5. Each subgroup had varied drug sensitivity and reached maximum divisibility, thus representing distinct pharmacological subtypes. C1 denotes the most sensitive and C5 the most resistant subtype to the drug Refametinib. Except for C3 where both sensitive and resistant cells were mixed, the other subgroups contained the homologous sensitive or resistant cells (Figure 1B). There were much more sensitive cells in C1 and C2 than resistant cells in C4 and C5 (Figure 1C). We then investigated how histological subtypes were distributed in the pharmacological subtypes (Figure 1D). C1 was composed of 25 histological subtypes, with top 2 cancers, SKCM (19.7%) and COREAD (12.4%). C5 was composed of 22 histological subtypes, with leading cancers including SCLC (31.8%) and BRCA (17.0%).

Our analysis established 367 pharmacological trees. The number of pharmacological subtypes with distinct drug sensitivities varied from 2 to 9 across 367 trees (Figure 1E). This suggested that heterogeneity of treatment effects across histological subtypes existed widely, and one pharmacological subtype presenting concordant drug response indeed comprised histological disparate cells to some extent. A high degree of treatment heterogeneity, meaning more than seven pharmacological subtypes, was observed for 54.0% (16.9% of the drugs had 7; 15.8% of the drugs had 8; and 21.3% of the drugs had 9 pharmacological subtypes) of the drugs. These drugs included Dabrafenib (BRAF inhibitor), Selumetinib (MEK1/2 inhibitor), Erlotinib (EGFR inhibitor), and Alectinib (ALK inhibitor). 44.1% of the drugs had 5 or 6 subtypes, showing a moderate degree of treatment heterogeneity, while 1.9% of the drugs had subtypes lower than 4, showing a low degree of heterogeneity related to drug therapy (Figure 1E).

We also particularly examined how pharmacological subtypes were constituted within the same histological tumor subtypes, including LUAD, BRCA, SKCM, SCLC, COREAD, and HNSC, in which the average number of cell lines per drug was more than 35 (Figures 1F–H). We built approximately 310 pharmacological trees for each type of the above cancers. The density distribution of the number of pharmacological subtypes indicated the varied treatment heterogeneity across 6 histological subtypes (Figure 1F). The shape of the distribution curve in LUAD was characterized by double kurtosis and significantly positively biased, and oppositely the curve in HNSC was negatively biased, suggesting the highest treatment heterogeneity in LUAD and lowest treatment heterogeneity in HNSC. Furthermore, K-S test was applied to evaluate if two different histological subtypes had the same level of treatment heterogeneity by comparing their cumulative distributions. The results showed the cumulative distribution curve of LUAD is significantly different from that of HNSC (*p = 1.11e-16*, K-S test) (Figure 1G). The pairwise comparisons between the curves of any two histological subtypes showed BRCA, SCLC, and SKCM had the statistically similar distributions (*p > 0.05*, K-S test), and other cancers had not (*p < 0.05*, K-S test) (Supplementary Figure S1).

To observe the varied treatment heterogeneity of the different histological subtypes in detail, the number of pharmacological subtypes of drugs across 6 histological cancers were shown in the heatmap (Figure 1H). Six histological tumors were arranged in columns according to their treatment heterogeneity, from high to low. Interestingly we observed that EGFR inhibitors (Afatinib, Cetuximab, and Gefitinib) targeting in EGFR signalling pathway (green drugs in Figure 1H) were clustered together (*p* < 0.001, Fisher’s exact test). And in LUAD and HNSC there were more pharmacological subtypes of EGFR inhibitors than other drugs, suggesting high treatment heterogeneity for EGFR inhibitors in LUAD and HNSC. In addition, drugs targeting ERK/MAPK signalling (red drugs in Figure 1H) were clustered together (*p* < 0.001, Fisher’s exact test). There was high treatment heterogeneity for these drugs in SKCM.

### Genomic Alterations Associated With Pharmacological Subtypes

**FIGURE 2:**
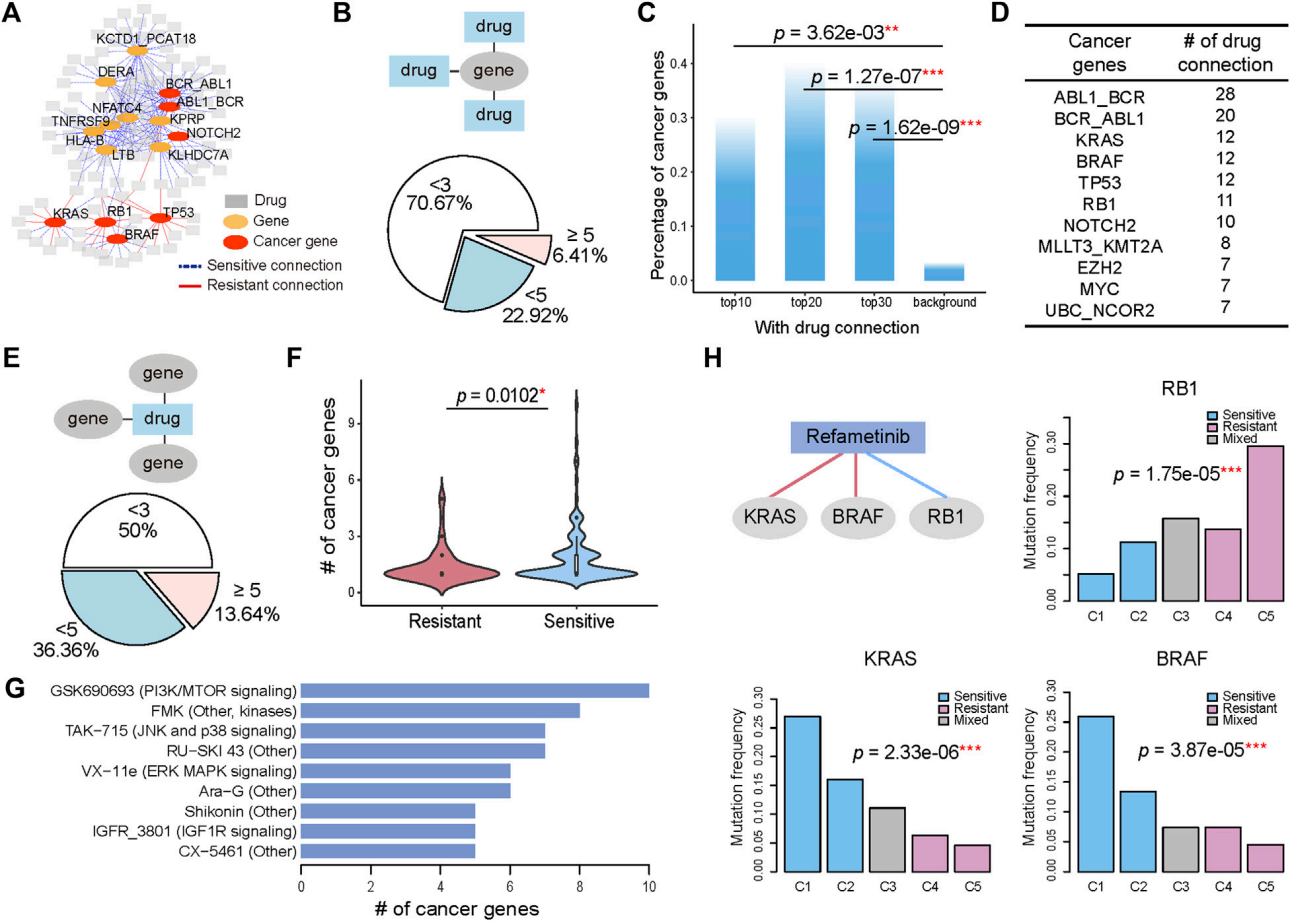
Genomic alterations determine the various drug sensitivities across pharmacological subtypes. **(A)** A gene with its genomic alteration significantly changed across the pharmacological subtypes of a drug is linked with the drug, generating a gene-drug network. A sensitive connection means that subtypes with high drug sensitivity have a more frequent occurrence of genomic alterations. Conversely, those that are less frequent are defined as resistant connections. The 15 hub genes with most connections to drugs are shown. **(B)** 6.41% genes have more than 5 connected drugs. In addition, 70.67% of genes have fewer than 3 connected drugs. **(C)** The percentage of cancer genes in the top 10, 20, and 30 genes, and the genome-wide 21,972 genes are referred to as background. **(D)** The top cancer censor genes defined by COSMIC and the corresponding number of drug connections. **(E)** When considering the drugs connected to the genes, 13.64% of drugs have more than 5 connected cancer genes. In addition, 50% of drugs have fewer than 3 connected cancer genes. **(F)** The number of genes with sensitive connections is significantly greater than the number of genes with resistant connections. **(G)** Drugs with cancer gene connections greater than 5 were ranked. **(H)** BRAF mutation, RB1 mutation and KRAS mutation are associated with the five pharmacological subtypes of Refametinib. Histograms show the mutation ratio of the three genes in the five pharmacological subtypes of Refametinib.

To identify the molecular alterations associated with varied drug sensitivity, we examined whether the genomic alterations, including mutations and translocations, significantly changed across the pharmacological subtypes. The statistical significance was determined by *p* values calculated with the Chi-squared test and corrected with the Benjamini-Hochberg method for controlling the false discovery rate. If the pharmacological subtypes with higher drug sensitivity had more frequent mutation or translocation events, we defined their association as a sensitive connection. If the opposite was true, we defined their association as resistant. Consequently, genes with specific genomic alterations were associated with the respective drugs. Since one gene could have multiple connected drugs and one drug could have multiple connected genes, these gene-drug connections ultimately composed a network that allowed us to investigate the contribution of either a drug or gene to the holistic understanding of how altered genes connect to drug responses (Figure 2A). The hub genes with the most connections to the drugs, including known cancer genes KRAS, RB1, BRAF, TP53, NOTCH2, and other genes LTB, TNFRSF9, DERA et al. were highlighted in the network.

We first investigated the distribution of genes on their connected drugs (Figure 2B). Our analysis identified 624 genes whose genomic alterations were connected with at least two drugs’ pharmacological subtypes. Most of the genes (70.67%) had fewer than 3 connections with drugs, but 40 (6.41%) genes whose genomic alterations were associated with more than 5 drugs. By ranking genes by their number of connections to drugs, we found that the top ranked genes were enriched in cancer genes (Figure 2C; Supplementary Figure S2), a catalogue of genes with mutations or fusions that are causally implicated in cancer provided by the COSMIC database (Tate et al., 2019). The top 10 genes connected to more than 12 drugs, 30% of which were remarkably annotated as cancer genes (Figure 2C). These cancerous percentages for the top 10 (*p* = 3.62e-03, Fisher’s exact test), top 20 (*p* = 1.27e-07, Fisher’s exact test), and top 30 genes (*p* = 1.62e-09, Fisher’s exact test) were significantly higher than the background when using genome-wide 21,972 genes as a reference (Figure 2C). Among the top ranked oncogenes, ABL1_BCR ranked first with connections to 28 drugs, followed by BCR_ABL1 and KRAS with 20 and 12 drugs (Figure 2D), implying that the highly drug-connected genes that are not currently identified as cancer genes could actually be causally implicated in cancer therapy, such as KCTD1_PCAT18 fusion, TNFRSF9, LTB, and NFATC4, which showed connections to over 18 drugs and ranked as the top five genes (Supplementary Figure S2).

We then investigated the distribution of drugs on their connected genes. In this analysis, we focused only on cancer censor genes. For the majority of drugs (86.36%), the number of connected genes ranged from 2 to 5 (Figure 2E). Among the pharmacological subtypes, genes whose alteration was sensitively connected with drug resistance were significantly more abundant than those that were connected in a resistant manner (*p* = 0.0102, student *t* test). (Figure 2F). We ranked the drugs with more than 5 genes’ connection with their pharmacological subtypes (Figure 2G). Drugs with gene connections greater than 5 included GSK690693, affecting the PI3K/MTOR signaling pathway, TAK−715, affecting the JNK and p38 signaling pathway, VX−11e, affecting the ERK MAPK signaling pathway, and IGFR_3801, affecting the IGF1R signaling pathway.

Next, we observed how the mutation rate of genes associated with the variability of drug sensitivity changed among drug-sensitive and drug-resistant subtypes. Taking the MEK1/2 inhibitor Refametinib as an example, three genomic alterations were found to connect to the drug and be associated with pharmacological subtypes. As shown in Figure 2H, BRAF mutations and KRAS mutations occurred significantly more frequently (over 25%) in the sensitive subtype C1 and then decreased gradually in the subtypes C2, C3, C4, and C5. Here one subtype C3 with sensitive and resistant cells mixed was excluded in the analysis. Conversely, RB1 mutation, occurred more frequently in the resistant subtypes. Therefore, C1 group, consisting mainly of SKCM (19.7%) and COREAD (12.4%) (Figure 1D), was significantly sensitive to Refametinib due to its high occurrence of BRAF and KRAS mutations and low occurrence of RB1 mutations (Figure 2H). Other types of MEK inhibitors, including PD0325901, Trametinib, Selumetinib, and CI-1040, further confirmed that BRAF, KRAS, and RB1 mutations were robustly connected to pharmacological subtypes, contributing to the drug sensitivity of MEK inhibitors (Supplementary Figure S3). Our analysis revealed that the varied distribution of genomic alterations across pharmacological subtypes could lead to their differences in response to anticancer therapies.

### Expression Alterations Associated With Pharmacological Subtypes

**FIGURE 3:**
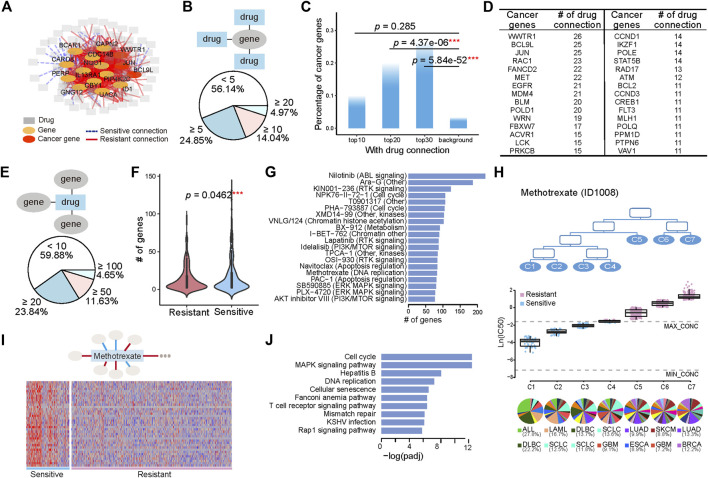
Tissue-specific gene expression determines the various drug sensitivities across pharmacological subtypes. **(A)** A gene with its expression significantly changed across the pharmacological subtypes of a drug is linked with this drug, generating a gene-drug network. A sensitive connection means that subtypes with high drug sensitivity have higher gene expression. Conversely, those with low expression are defined as resistant connections. The 15 hub genes with the most connections to drugs are shown. **(B)** Pie chart illustrates the numerical proportion of the number of connected drugs with genes whose expression is associated with the pharmacological subtypes. A total of 4.97% of genes have more than 20 connected drugs. In addition, 56.14% of genes have less than 5 connected drugs. **(C)** The percentage of cancer genes in the top 10, 20, and 30 genes, and the genome-wide 21,972 genes are referred to as background. **(D)** The top cancer censor genes defined by COSMIC and the corresponding number of drug connections. **(E)** Distribution of the genes functioning in the core cancer pathways whose expression is connected with pharmacological subtypes. A total of 4.65% of drugs have more than 100 connected genes. **(F)** The number of genes with sensitive connections is significantly greater than the number of genes with resistant connections. **(G)** Top 20 drugs with the most connections to genes. **(H)** Seven subtypes, C1, C2, C3, C4, C5, C6, and C7, have varied drug sensitivity to methotrexate, with C1 being the most sensitive to the drug and C7 being the most resistant to the drug. The boxplot below shows the drug sensitivities of the three subtypes (leaf nodes) C1, C2, C3, C4, C5, C6, and C7, indicating a gradually increasing degree of resistance to the drug. **(I)** A total of 38 genes are highly expressed in sensitive group (C1, C2, and C3) and lowly expressed in resistant group (C5, C6, and C7). **(J)** Pathways enriched by the 38 highly expressed genes in sensitive group (C1, C2, and C3).

We also associated mRNA expression with the pharmacological subtypes. The connection between gene expression and pharmacological subtypes was determined by *p* values calculated with the student *t* test and corrected with the Benjamini-Hochberg method for controlling the false discovery rate. If the subtypes with higher drug sensitivity had higher gene expression, we defined this connection as sensitive. If the opposite was true, we defined the connection as resistant. The connections between genes and drugs constituted a network (Figure 3A). To narrow down the genes whose expression levels were directly associated with drug sensitivity, we only kept genes which act in core cancer pathways (Supplementary Table S1), and whose Pearson correlation between drug sensitivity and gene expression were greater than 0.3 in the sensitive subtypes. The hub genes with the most connections to the drugs, including known cancer genes WWTR1, JUN, BCL9L and other genes NQO1, CAPN2, and PERP et al., were highlighted in the network.

We first investigated the distribution of genes on their connected drugs (Figure 3B). Our analysis identified 684 genes whose expression was connected to at least two drugs’ pharmacological subtype. Among them, 4.97%, 14.04%, and 24.85% of genes respectively had connections with greater than 20, 10, and 5 drugs. The top 30 genes connected to more than 20 drugs, 25% of which were annotated as cancer genes (Figure 3C; Supplementary Figure S4). These cancerous percentages for the top 20 (*p* = 4.37e-06, Fisher’s exact test), and top 30 genes (*p* = 5.84e-52, Fisher’s exact test) were significantly higher than the background. The top genes with their expression alterations associated with subtypes were different from those with genomic alterations associated with subtypes (Figures 2D, 3D). Among them, WWTR1, BCL9L, and JUN ranked the top three with connections to 26 drugs and 25 drugs (Figure 3D).

We then investigated the distribution of drugs on their connected genes. The number of genes whose expression was associated with the subtypes varied from 0 to 223 across the drugs, with 4.65% drugs had more than 100 gene connections (Figure 3E). Comparing with genomic alterations, there were more genes whose expression was connected to the drugs. Moreover, genes whose expression was sensitively connected with drug resistance across pharmacological subtypes were significantly more abundant than genes that were resistant connected (*p* = 0.0462, student *t* test) (Figure 3F). The top 20 drugs with the most connections to genes included Nilotinib, affecting the ABL signaling, PHA−793887 and NPK76−II−72−1, affecting cell cycle, Lapatinib and KIN001−236, affecting RTK signaling pathway, Navitoclax, affecting Apoptosis regulation, and some compounds such as Ara−G, XMD14−99 et al., affecting multiple or unknown pathways (Figure 3G).

Methotrexate, as an example, is a chemotherapy that specifically acts during DNA and RNA synthesis, and cancer cells were classified into seven subtypes (Figure 3H) in terms of drug sensitivity to methotrexate. The first subtype, C1, was most sensitive to the drug, and the seventh subtype, C7, was the most resistant to the drug (Figure 3H). C1 included 12 histological subtypes, with the top 2 shown in the bottom of Figure 3H, ALL and DLBC. C2 and C3 were composed of 18 and 19 histological subtypes, respectively, with SCLC both included in the top 2 (Figure 3H). A total of 38 genes with high expression in sensitive group (C1, C2, and C3) and low expression in resistant group (C5, C6, and C7), which had sensitive connections to pharmacological subtypes of methotrexate, were selected (Figure 3I). Pathway enrichment analysis showed that these genes were enriched in pathways including cell cycle and DNA replication et al. (Figure 3J), suggesting that the ectopic activation of cell cycle led to the sensitive response of ALL, DLBC, LAML, and SLCL to Methotrexate.

### Redefining the Similarity of Cells Based on Pharmacological Subtypes

**FIGURE 4:**
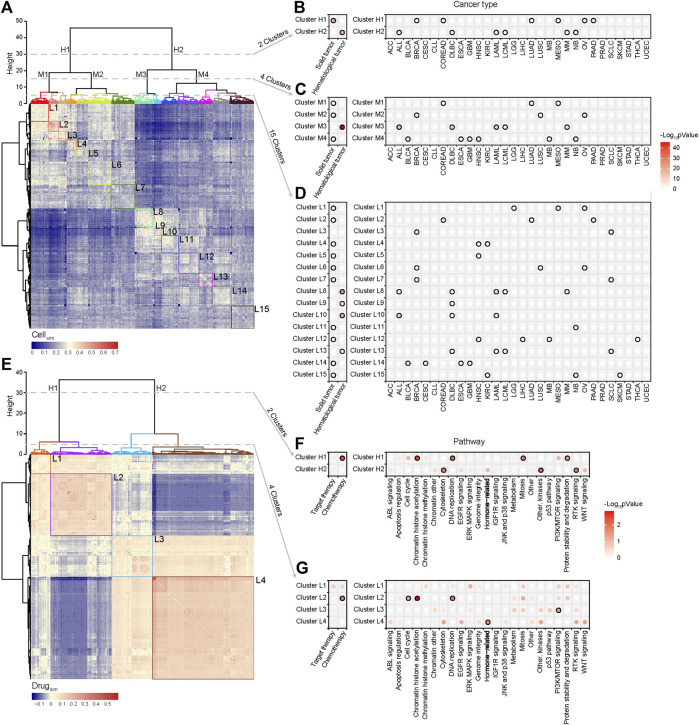
Cell similarity and drug similarity based on pharmacological subtypes. **(A)** Hierarchical clustering of a cell similarity matrix which was generated based on drug response of cells to drugs, with the red color indicating high similarity, and the blue color indicating low similarity between two cell lines. Clusters were formed by partitioning of the dendrogram at a high, median and low height respectively, which indicates the distance between the clusters. **(B)** Two clusters (H1, H2) were formed by cutting the dendrogram at a height of 30. Points with black border represent statistical significance (*p* < 0.05) determined by Fisher’s exact test. Almost all histological subtypes of blood cancer were grouped together. **(C)** Four clusters (M1, M2, M3 and M4) were formed by cutting the dendrogram at a height of 15. **(D)** Fifteen clusters (L1-15) were formed by cutting the dendrogram at a height of 5. Cancers with the same tissue of origin and organ did not cluster together. **(E)** Hierarchical clustering of the drug similarity profiles with the red color indicating a high similarity and the blue color indicating a low similarity. Clusters were formed by partitioning of the dendrogram at a high and low height respectively. **(F)** Two clusters (H1, H2) were formed by cutting the dendrogram at a height of 30. Points with black border represents statistical significance (*p* < 0.05) determined by Fisher’s exact test. Chemotherapy related drugs were grouped together. **(G)** Four clusters (L1, L2, L3, and L4) were formed by cutting the dendrogram at a height of 5. Cytoskeleton, EGFR signalling, Hormone-related and WNT signalling pathways cluster together.

Pharmacological subtypes provide us a new point of view to redefine the similarity of cells purely from therapeutic concordance. Thus, we calculated the similarity of pairwise cells based on pharmacological subtypes of drugs. If there are quite a few cases that two cells are in the same sensitive or resistant pharmacological subtypes of drugs, these cells gain a high similarity. Otherwise, they gain a low similarity. The similarity of cells defined a hierarchical cluster as shown in Figure 4A. We further applied the Fisher’s exact test to observe if the cells in the same clusters were from the same histological subtypes (Figures 4B–D). The points with black border in the figure represent statistical significance (*p* < 0.05), with the smaller *p* values shown in red.

The results showed that the cancer cell lines were respectively divided into 2, 4, and 15 clusters at the different hierarchical cluster levels (Figure 4A). Two clusters at the high level were reflected by two distinct patterns of similarity (Figure 4A). One cluster (Cluster H1) was significantly correlated with solid tumors including BRCA, COREAD, LUAD, MESO, OV, and PAAD, and the other (Cluster H2) hematological tumors including ALL, DLBC, LAML, LCML, and MM (Figure 4B). Here, the point with black border represents statistical significance (*p* < 0.05, Fisher’s exact test) of the association between the cluster and cancer type. So in a holistic view, the different types of hematological tumors shared a similar drug sensitivity profile, which was quite different from the profile of solid tumors. However, for a given cancer type (the right panel of Figure 4B), such as ACC, BLCA, CESC, CLL, or ESCA et al., its cells were unbiasedly distributed in two clusters (*p* > 0.05, Fisher’s exact test), showing the treatment heterogeneity for the cells from the same tissue origin. At the median hierarchical levels, 4 clusters were identified (Figure 4C). Among them, Cluster M3 was specifically associated with haematological tumors (*p* < 0.001, Fisher’s exact test) including ALL, DLBC, LAML, LCML, and MM. The treatment heterogeneity was still observed in cancers.

When cell lines were divided into 15 clusters at the lower hierarchical level (Figure 4D), we observed that the cells from ACC, CLL, STAD, or UCEC were unbiasedly distributed in the different clusters, still showing their treatment heterogeneity. Interestingly, the cells from some cancers such as HNSC, KIRC, and SCLC were enriched in more than 2 clusters with distinctly different drug response. For instance, HNSC cells were enriched in Clusters L12, L4 and L5; KIRC cells were enriched in Clusters L15 and L4; SCLC cells were enriched in Clusters L13, L3, and L7. Interestingly, the SCLC cells in Cluster L13 shared a more similar profile with hematological tumors including DLBC, LAML, and LCML. To investigate the underlying mechanism of such treatment heterogeneity in SCLC, we applied DESeq2 analysis to call the differentially expressed genes between Cluster L13 and Cluster L3, and further identified the differential functions via gene set enrichment analysis (GSEA) (Subramanian et al., 2005). The results showed that the functions like DNA replication, RNA transport et al. were significantly upregulated in Cluster L13 cells, while in Cluster L3 cells the functions like immune response, antigen processing and presentation et al. were mainly upregulated (Supplementary Figure S5A). Moreover, MYC, as an oncogenic transcription factor of cell growth and proliferation via enhancing the cell cycle regulated genes (Oster et al., 2002), was significantly upregulated in Cluster L13 than in L3, with a log2-fold change of 4.28 (Supplementary Figure S5B). These suggested that SCLC cell lines in Cluster L13 were higher Myc-dependent than those in Cluster L3, characterized with increased cell proliferation and evasion of immune response.

### Redefining the Similarity of Drugs Based on Pharmacological Subtypes

The similarity of pairwise drugs was also defined as the summarized similarity of drug response across the same cells which were tested for both drugs. For two drugs, if there are quite a few of cell pairs which are in the sensitive (or resistant) pharmacological subtypes of one drug are also in the sensitive (or resistant) subtypes of the other drug, they gain a high similarity. Otherwise, they gain a low similarity. We then generated a drug similarity profile across 367 drugs, and similarly, a hierarchical clustering algorithm was used to group drugs based on drug similarity (Figure 4E). The results showed that at the high level, drugs were divided into two clusters, demonstrating two different similarity patterns. The first cluster (Cluster H1) mostly included chemotherapy drugs such as those effecting in DNA replication and mitosis, and the second cluster was mixed with target and chemotherapy drugs, specifically enriched by the drugs on RTK signaling, other kinases, and cytoskeleton (Figure 4F). When the drugs were divided into 4 clusters (Figures 4E,G), we observed that the drugs enriched in L2 included chemotherapy drugs effecting in cell cycle, chromatin histone accelylation, and DNA replication, the drugs enriched in L3 were functioning in PI3K/mTOR signaling pathway, and the drugs enriched in L4 were hormone related ones. Moreover, L4, accounting for a massive part of drugs, also included the drugs targeting the different types of signaling pathways such as ABL, EGFR, Jak, and P38, RTK, and WNT signalling. These drugs did not reach the significant level but provided the obvious association.

### An Access to Pharmacological Subtypes of Drugs

To enable knowledge sharing and reuse, we developed a database (http://www.hywanglab.cn/dtdb/, Supplementary Figure S6), which provided the comprehensive knowledge of pharmacological subtypes of drugs in this study, for supporting the further exploration of therapeutic response-based classification of tumors. The web interface of the database is user-friendly and allows users to search, browse and download data. A record of the database includes the following information: the pharmacological subtypes of a drug, the drug sensitivity values of each subtype, the distribution of histological subtypes in each pharmacological subtype, genomic and expression alterations connected to subtypes. In addition, the users can acquire the other information of a drug, a gene, or a specific cancer type in terms of our analysis. For example, given a drug, the other drugs with the most similar drug response across cancer cells are provided. Similarly, given a cancer cell line, the other cell lines with the most similar drug response across drugs are provided. Given a histological cancer type, such as SCLC, the pharmacological subtypes with the distinct treatment response were depicted, as well as the underlying mechanism of such treatment heterogeneity. The resources are expected to support the researches of precision medicine from a purely therapy-oriented perspective.

## Discussion

In this study, we integrated drug response information to pharmacologically reclassify tumor subtypes, as well as identify intrinsic concordant molecular mechanisms. Unlike the many studies that have aimed to unravel cancer heterogeneity by defining subtypes within the same tissues, our study aimed to systematically uncover that cancers across disparate tissues of origin can belong to one pharmacological subtype, benefiting from similar anticancer therapies, because they share a common molecular mechanism of oncogenesis. Besides, we also developed the new measures to redefine cell similarity and drug similarity from the therapeutic concordance, which provided a new point of view to study cancer heterogeneity. The similarity of cells further depicted that cells from the different origin of tissue could share the similar responses of drugs; likewise, that cells from the same origin of tissue could have distinct drug responses, thus indicating the new subtypes.

Our analysis identified, for instance, that although SKCM and COREAD are histologically different, both belonged to one pharmacological subtype, the MEK1/2 inhibitor C1 subtype, and were similarly sensitive to MEK1/2 inhibitors (Figures 1A–D). By connecting genomic alterations with pharmacological subtypes, mutations in the oncogene BRAF or KRAS were found to be overwhelmingly more frequent in the C1 group (Figure 2H). BRAF and KRAS are the kinases upstream of MEK1/2 that transmits the signals down through MEK1/2 without other major signalling branches (McCubrey et al., 2007; Simanshu et al., 2017). Our results illustrated that MEK inhibitors were able to effectively block aberrantly activated signals from BRAF or KRAS mutations. Further investigation by cBioportal (https://www.cbioportal.org/) of TCGA patients also revealed that approximately 60% of THCA (thyroid carcinomas) and 50% of SKCM (melanomas) harboured BRAF mutations, and 65% of PAAD (pancreas) and 40% COREAD (colorectal carcinomas) possessed KRAS mutations, a rate that was strikingly higher than that of other cancers (Supplementary Figures S7A,B). Given the high mutant frequency of BRAF in THCA and SKCM, and KRAS in PAAD and COREAD, such 4 cancer types were significantly enriched in C1 pharmacological subtype of MEK1/2 inhibitor Refametinib (THCA, *p* = 1.90e-2; SKCM, *p* = 1.23e-14; PAAD, *p* = 3.01e-4; COREAD, *p* = 3.84e-5; Hypergeometric Distribution Test) (Supplementary Table S2). In contrast, mutations in the oncogenes RB1 was found to be less frequent in the C1 group than in other groups. RB1 is a negative regulator of the cell cycle, with its active hypophosphorylated form binding the transcription factor E2F1 (Chen et al., 2009). RB1 mutations lead to the ectopic activation of the cell cycle, which cannot be controlled using MEK inhibitors. This may be because RB1, located downstream of MEK1/2, gain ectopic activation independent of upstream stimulation. In another instance, therapeutic concordance was identified across histologically disparate blood cancers in regard to methotrexate, a chemotherapy drug specially acting during DNA and RNA synthesis (Figure 3H). This is well known that Methotrexate is used in haematological malignancies but less so in solid cancers (Koźmiński et al., 2020). Functional analysis of the sensitively correlated genes showed that haematological tumors were ectopically activated in cell cycle than solid tumors (Figures 3I,J). The molecular basis of such extraordinary activation of the cell cycle in haematological tumors could explain their good response to chemotherapy drugs, which work mainly by inhibiting mitosis and cell division. This demonstrates pharmacological subtypes benefiting from similar anticancer therapies can be due to common molecular mechanisms related to tissue-specific gene expression and pathways.

We also investigated the treatment heterogeneity for six types of histological cancers, including LUAD, BRCA, SKCM, SCLC, COREAD, and HNSC. High treatment heterogeneity in LUAD was observed compared with other types of cancers, suggesting that anticancer therapies for LUAD should be more complicated than those for other types of cancers (Figures 1F–H). Moreover, the increased number of subtypes of EGFR inhibitors in LUAD, and of drugs targeting in ERK/MAPK signalling in SKCM, indicated there was high treatment heterogeneity when EGFR inhibitors were applied into the therapies of LUAD patients and when ERK/MAPK signalling targeting drugs were applied into SKCM patients (Figure 1H). We also observed the low treatment heterogeneity for HNSC (Figures 1F,G). However, our findings were only based on the current information provided by the CGP dataset. The low treatment heterogeneity could be due to the fact that active drugs in this histological cancer are potentially still missing. Drug development in future may increase the treatment heterogeneity as well.

Our analysis showed that genes whose genomic/expression alterations frequently connect to anticancer drugs had a higher likelihood of being cancer genes. At genomic level, the top 10 genes were connected to more than 10 drugs, 30% of which were annotated as cancer censor genes. At expression level, each of genes connected to more drugs and the top genes had higher cancerous percentages. This indicated that some genes currently not identified as cancer genes could be causally implicated in cancer. In our analysis, KCTD1_PCAT18, TNFRSF9, and LTB, as the top three most connected genes, were worth for further investigation. KCTD1 (potassium channel tetramerization domain containing 1) was reported to regulate the Wnt/β-catenin pathway (Li et al., 2014; Hu et al., 2020), and PCAT18 (prostate cancer-associated transcript 18, lncRNA), was found to promote the progression of colorectal or gastric cancer through miR-759 or miR-135b (Hu et al., 2020; Zhang et al., 2020), thus KCTD1_PCAT18 fusion may resulting in the development of cancer. TNFRSF9 (tumor necrosis factor receptor superfamily member 9), also known as 4-1BB and CD137, is an immune co-stimulatory receptor. TNFRSF9 is expressed on activated immune cells including natural killer (NK) cells, effector T cells and antigen presenting cells, among them dendritic cells, macrophages, and B cells (Fröhlich et al., 2020). LTB (Lymphotoxin Beta) is a type II membrane protein of the TNF family. Giuseppina et al. found that a decrease in lymphotoxin-β production by tumor cells was associated with a loss of follicular dendritic cell phenotype and diffuse growth of follicular lymphomas (Pepe et al., 2018). Recently, the gene was found to be associated with immune infiltration of breast and endometrial cancer tumors (Ding et al., 2020; Terkelsen et al., 2020). The analysis using the TCGA dataset showed that TNFRSF9 was altered in approximately 5% of cases of Cholangiocarcinoma, adrenocortical carcinoma (ACC) and lymphoid neoplasm diffuse large B-cell lymphoma (DLBC), with deep deletion being the dominant alteration (Supplementary Figure S8A). In addition, more than 14% of DLBC patients had altered LTB genes, with mutation and deletion being the major alterations (Supplementary Figure S8B). Therefore, the importance of the roles of these genes in cancers could be underappreciated.

Our analysis also showed that some drugs connected to many known cancer genes (Figure 2G). This suggested that these drugs are unspecific or the genes are important for many different pathways. So these drugs may not be ideal from a therapeutic perspective. Moreover, genes whose alterations were sensitively connected with drug resistance were significantly more frequent than those resistant connected (Figures 2F, 3F). A sensitive connection meant that genomic alterations or high expression indicated increased drug sensitivity. In this way, genes with a sensitive connection could be indicators of therapeutic efficiency for connected drugs. Genes with resistant connections could be potential biomarkers for developing new therapies to overcome the resistance of connected drugs. Our analysis suggests that most of the genes whose expressions are altered during cancer initiation and development (driver genes or not) are located in ectopically activated pathways that could be controlled by anticancer drugs. Only a proportion of altered genes are outside of the cellular pathways targeted by anticancer drugs, and their alterations may maintain the growth signals of cancer cells when anticancer drugs are used. Therefore, these genes may be potential therapeutic biomarkers for overcoming drug resistance to the connected drugs.

Based on pharmacological subtypes, the cell similarity and the drug similarity could be re-defined (Figure 4). The cells from the same origin of tissue can be dispersed in the different pharmacological subtypes, showing the completely different response to the same drug, because of their intrinsically molecular heterogeneity. For instance, HNSC, KIRC, and SLCL cells were separated into more than two clusters with distinct drug response (Figure 4D). Specifically, the SCLC cells with more malignant signatures, in particular, higher expression levels of MYC, upregulated pathways involving DNA replication and cell cycle and downregulated pathways on immune response, were far away from the other SCLC cells and close to hematological tumors, instead. Interestingly, by connecting to expression alterations, our analysis further confirmed that SCLC cells more like hematological tumors had the characteristic of the ectopic activation of cell cycle, which led to their sensitive to a chemotherapy Methotrexate (Figures 3H–J). Our pharmacological analysis has recognized a new subtype of SCLC cells, with the high expression of an oncogenic transcription factor MYC as a marker, can benefit from chemotherapy than other SCLC subtypes. This finding was consistent with the latest studies (Bian et al., 2020; Ireland et al., 2020). Therefore, the subtypes identified by re-classifying the same origin of tissue using pharmacological data are worth to be further investigated. In addition, our analysis of the drug similarity unraveled that the drugs were categorized into two major groups, wherein the chemotherapeutic drugs tended to share the similar pattern (Figures 4E,F).

There are several limitations to this study. We measured the similarity of cells/drugs on the scale of all drugs/cells, so the similarity at the local space might be underestimated. Moreover, there is lack of experimental validation for our findings. To overcome these limitations, further proof of concept studies that reclassifying tumors based on therapeutic responses independent of histology subtypes is warranted.

In summary, this study demonstrates how pharmacogenomic data can be used to systematically classify cancers in terms of response to various compounds and provides us with a purely therapy-oriented perspective to view tumor classifications independent of histology subtypes. Moreover, the knowledge of pharmacological subtypes of 367 drugs are available via our website, providing the resources for precision medicine in the perspective of therapeutic response-based re-classification of tumors.

## Data Availability

Publicly available datasets were analyzed in this study. This data can be found here: https://www.cancerrxgene.org/downloads/bulk_download.

## Glossary

ACC: Adrenocortical carcinoma
ALL: Acute lymphoblastic leukemia
BLCA: Bladder urothelial carcinoma
BRCA: Breast invasive carcinoma
CESC: Cervical squamous cell carcinoma and endocervical adenocarcinoma
CLL: Chronic lymphocytic leukemia
COREAD: Colon adenocarcinoma and rectum adenocarcinoma
DLBC: Lymphoid neoplasm diffuse large B-cell Lymphoma
ESCA: Esophageal carcinoma
GBM: Glioblastoma multiforme
HNSC: Head and neck squamous cell carcinoma
KIRC: Kidney renal clear cell carcinoma
LAML: Acute myeloid leukemia
LCML: Chronic myelogenous leukemia
LGG: Brain lower grade glioma
LIHC: Liver hepatocellular carcinoma
LUAD: Lung adenocarcinoma
LUSC: Lung squamous cell carcinoma
MB: Medulloblastoma
MESO: Mlistdefinitionesothelioma
MM: Multiple myeloma
NB: Neuroblastoma
OV: Ovarian serous cystadenocarcinoma
PAAD: Pancreatic adenocarcinoma
PRAD: Prostate adenocarcinoma
SCLC: Small cell lung cancer
SKCM: Skin cutaneous melanoma
STAD: Stomach adenocarcinoma
THCA: Thyroid carcinoma
UCEC: Uterine corpus endometrial carcinoma
CGP: The Cancer Genome Project
IC50: Half maximal inhibitory concentration
TCGA: The Cancer Genome Atlas Program
FDA: U.S. Food and Drug Administration
RTK: Receptor tyrosine kinase
MEK: Mitogen-activated protein kinase kinase.
