# Supplementary material for: Therapeutic Response-Based Reclassification of Multiple Tumor Subtypes Reveals Intrinsic Molecular Concordance of Therapy Across Histologically Disparate Cancers
Source: Front Cell Dev Biol. 2021 Nov 12;9:773101. doi: 10.3389/fcell.2021.773101 (PMC8632957; doi:10.3389/fcell.2021.773101)
Supplement: Supplementary file 2 [file DataSheet4.DOCX]

The expression data used is stored in the Nut cloud (www.jianguoyun.com), the download link is: https://www.jianguoyun.com/p/Dczi4fcQhZ7qCRjooo4E
