# Supplementary figures and images for: Therapeutic Response-Based Reclassification of Multiple Tumor Subtypes Reveals Intrinsic Molecular Concordance of Therapy Across Histologically Disparate Cancers
Source: Front Cell Dev Biol. 2021 Nov 12;9:773101. doi: 10.3389/fcell.2021.773101 (PMC8632957; doi:10.3389/fcell.2021.773101)

Figure S5

A

## KEGG enrichment (Cluster L13 vs. L3)

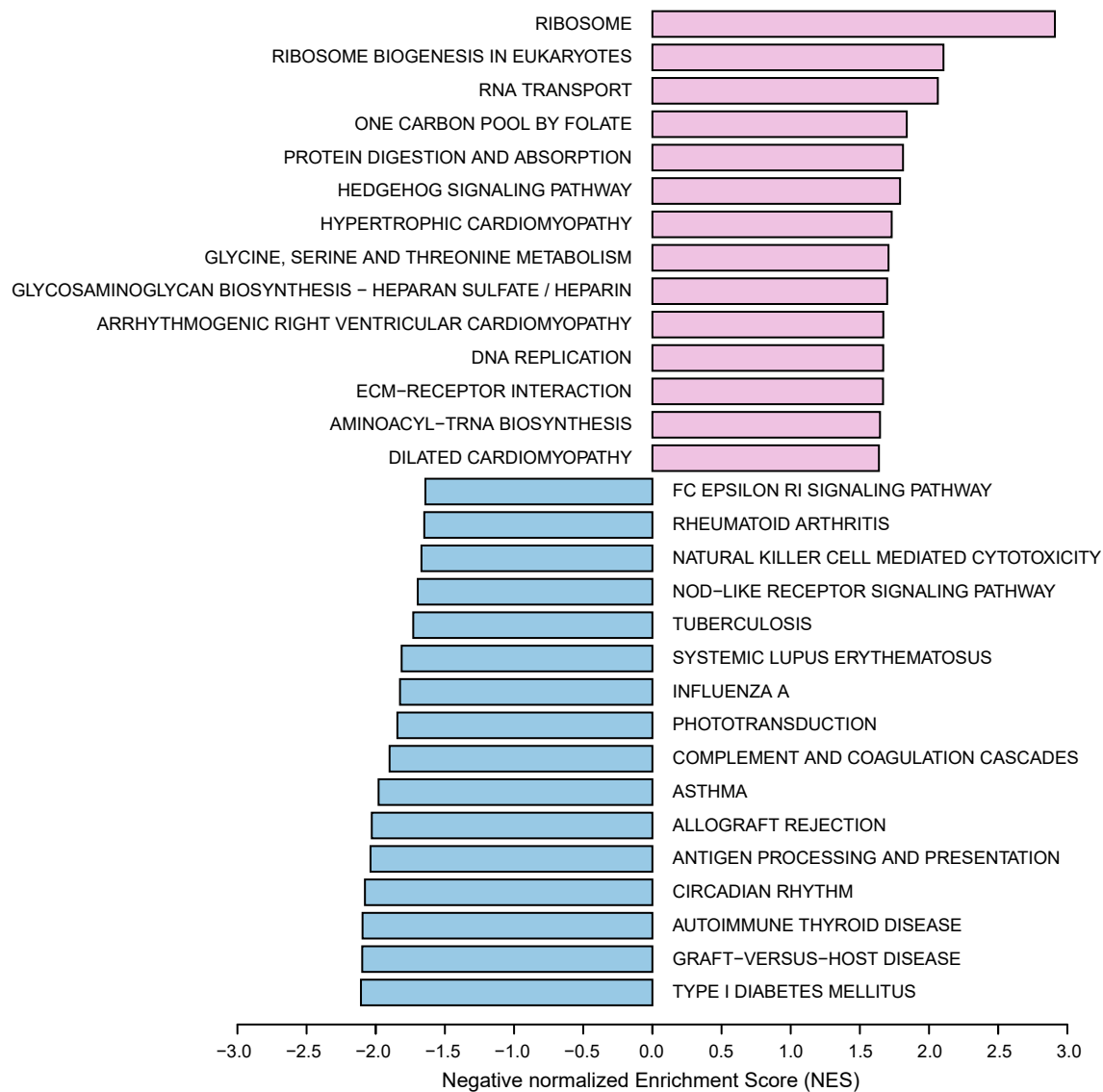

B

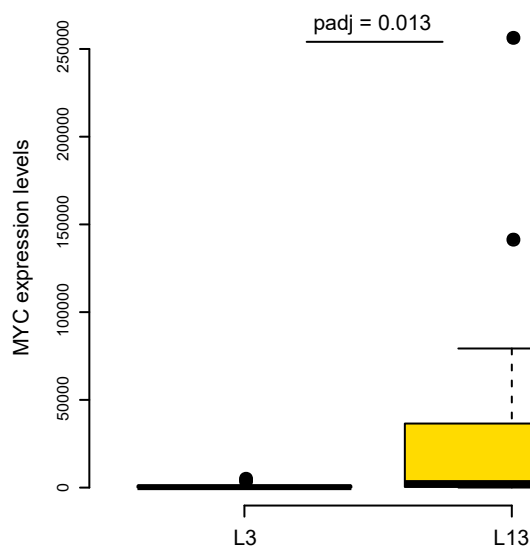

Supplement: Supplementary file 4 [file Image5.PDF]

Figure S8

A

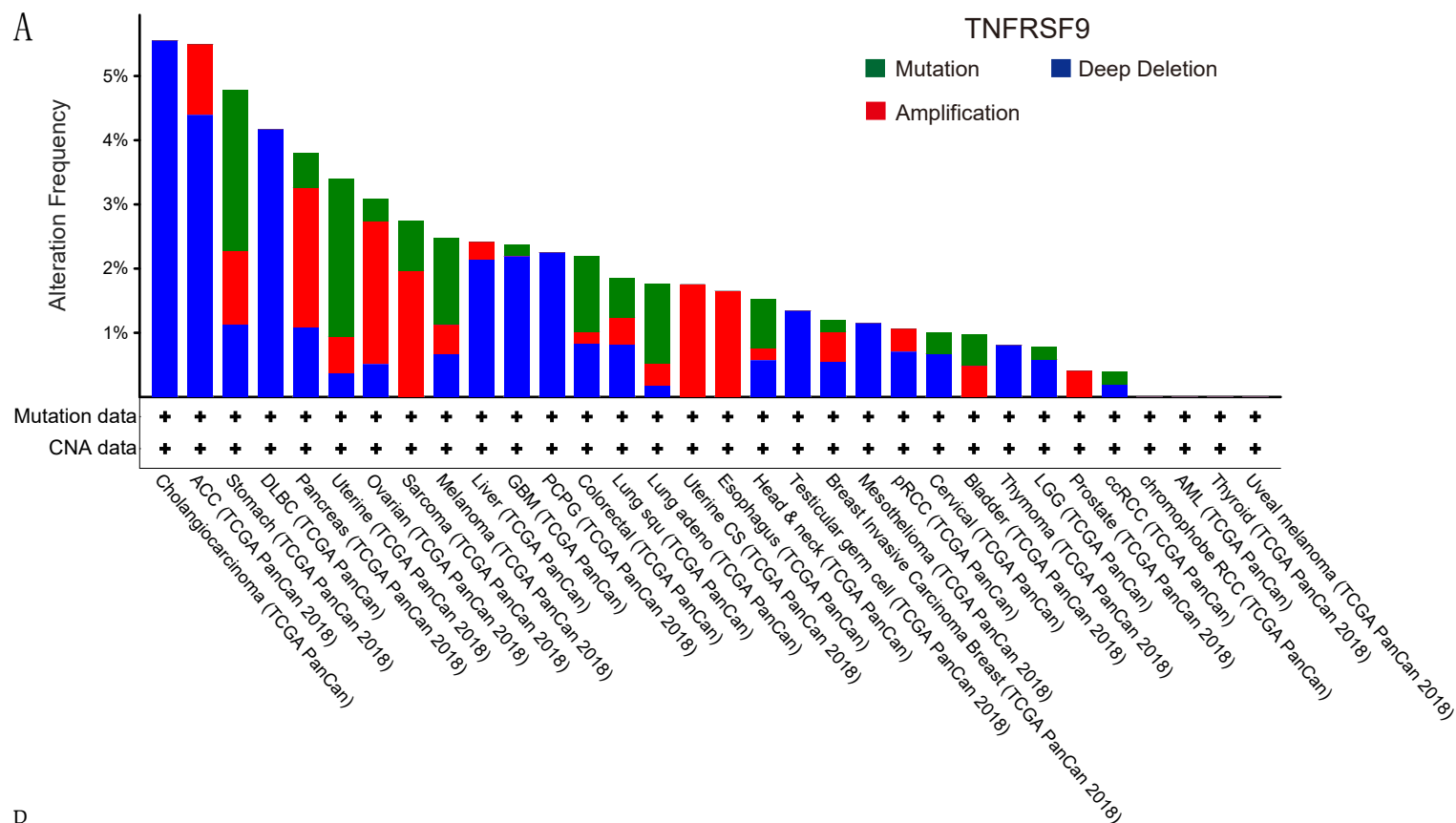

B

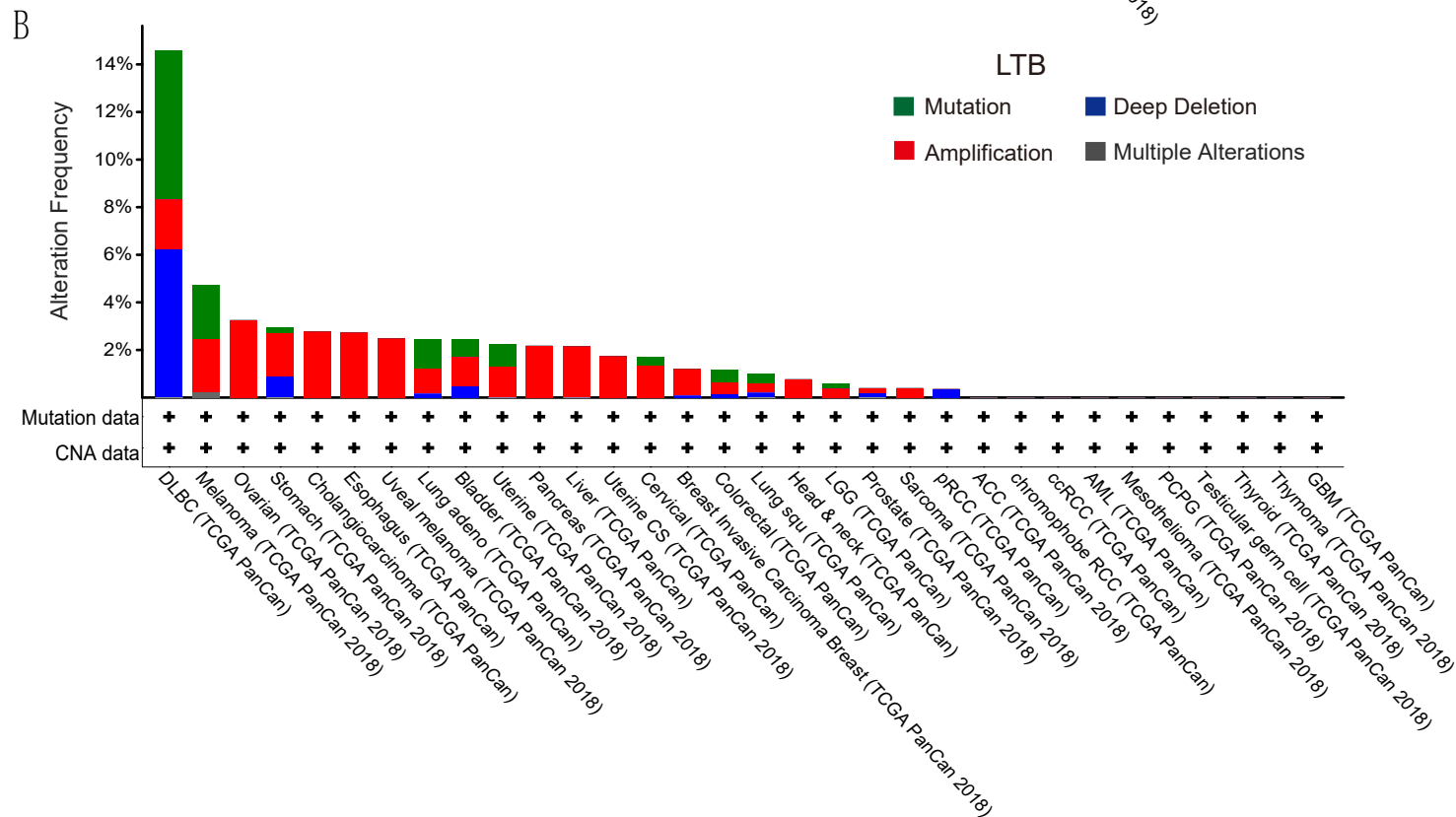

Supplement: Supplementary file 6 [file Image8.PDF]

Figure S3

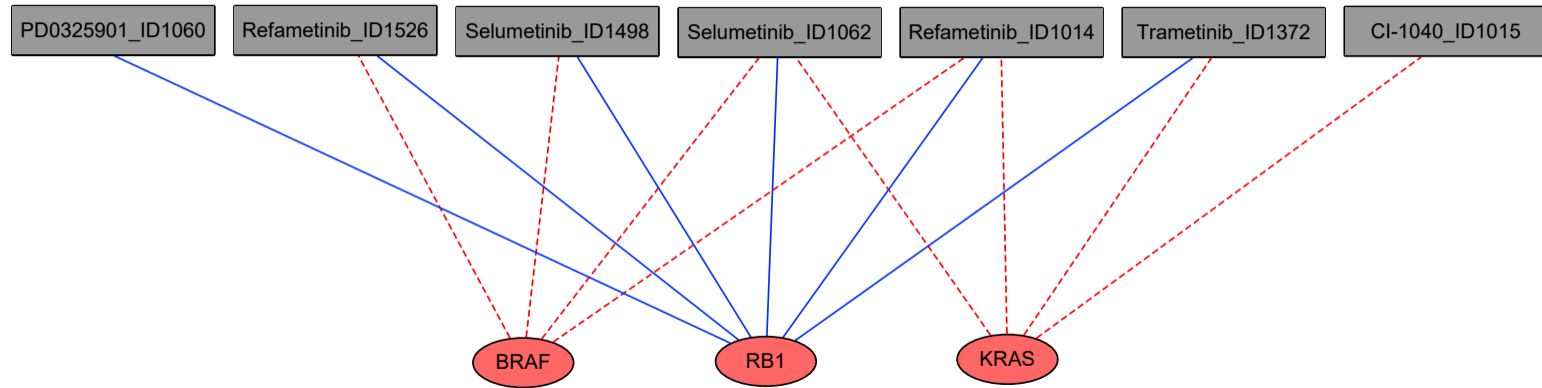

Supplement: Supplementary file 9 [file Image3.PDF]

# Figure S7

A

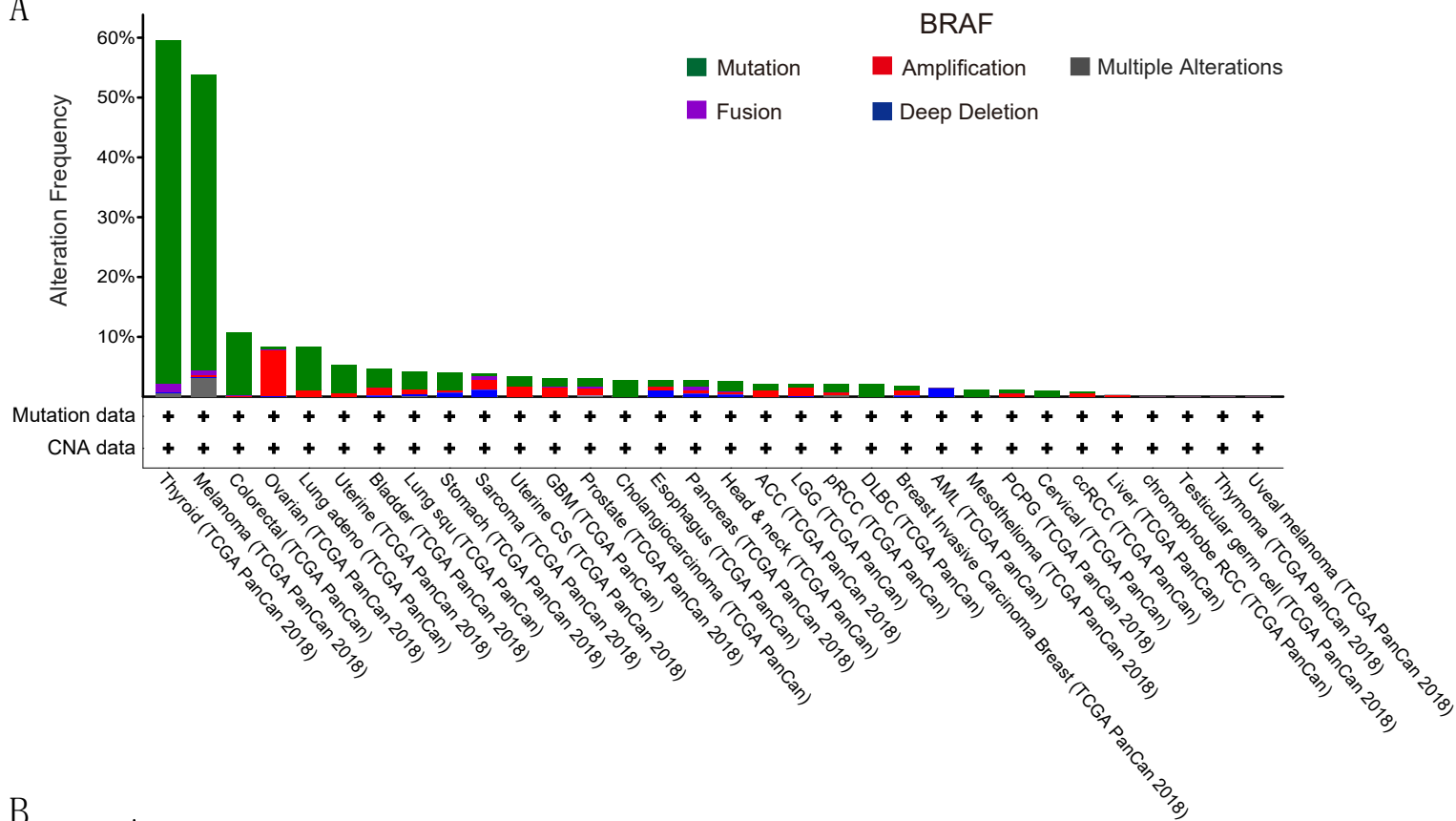

B

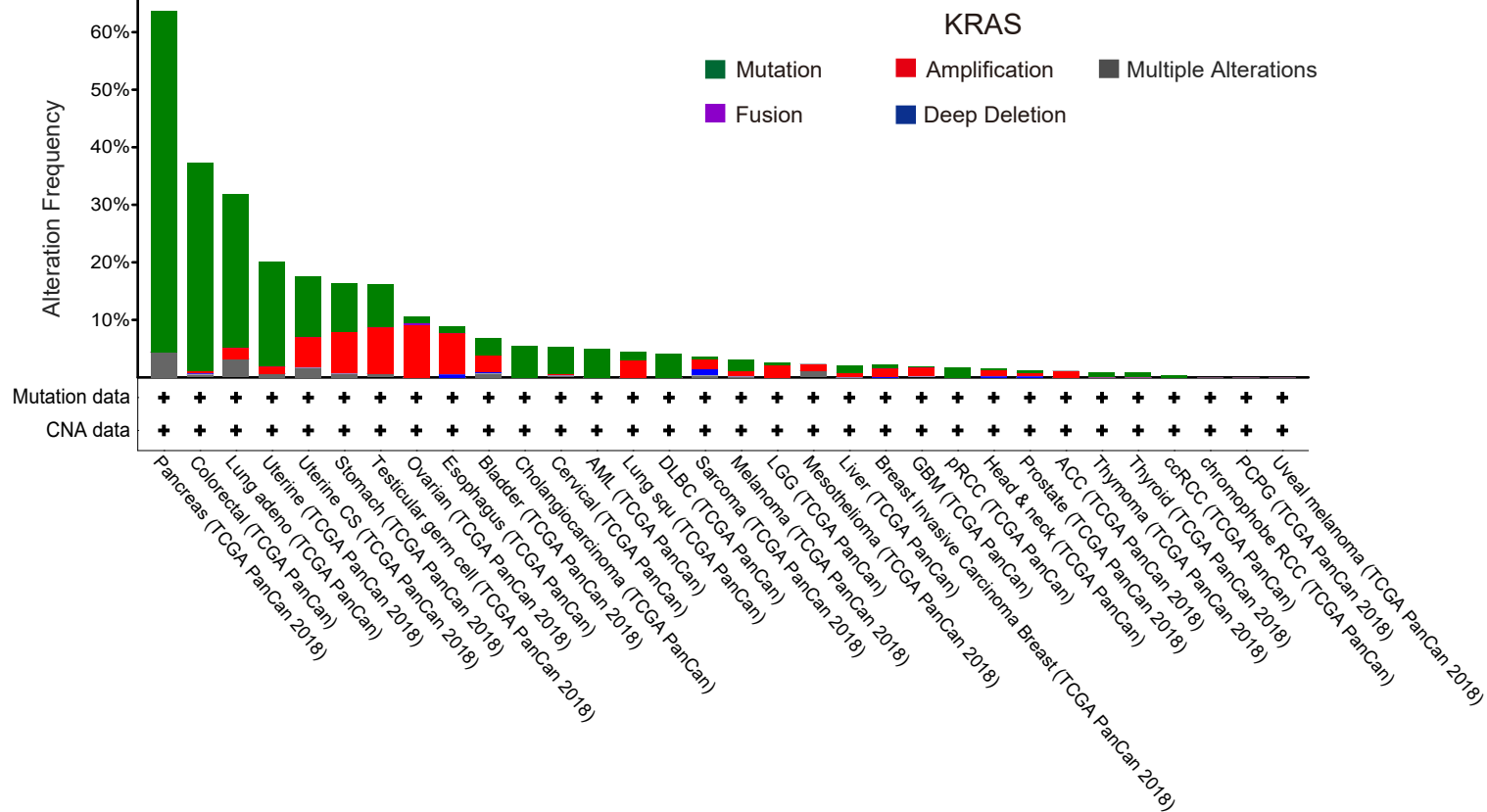

Supplement: Supplementary file 12 [file Image7.PDF]

Figure S1

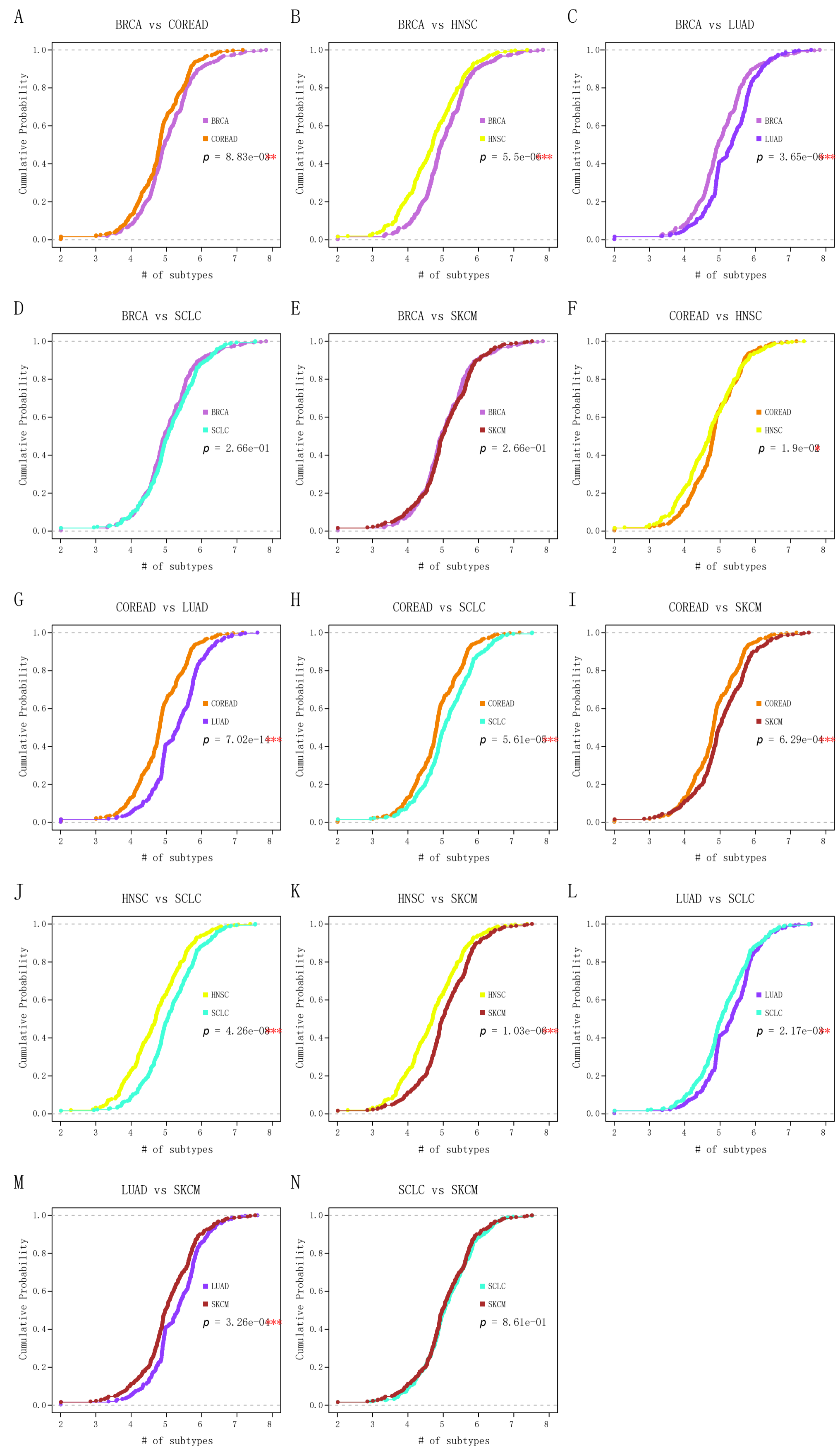

Supplement: Supplementary file 14 [file Image1.PDF]
