# Supplementary material for: Therapeutic Response-Based Reclassification of Multiple Tumor Subtypes Reveals Intrinsic Molecular Concordance of Therapy Across Histologically Disparate Cancers
Source: Front Cell Dev Biol. 2021 Nov 12;9:773101. doi: 10.3389/fcell.2021.773101 (PMC8632957; doi:10.3389/fcell.2021.773101)
Supplement: Supplementary file 5 [file Image6.PDF]

Figure.S6

Accessible Data

Cancer Genome Project

e.g.

- Expression profiles
- Mutation profiles
- Fusion profiles
- Drug response profiles

Pharmacological subtypes

e.g.

- Pharmacological subtypes of drugs
- Genomic/expression alterations connected to subtypes
- The distributions of histological cancers across subtypes

Similarity of drugs, cancer cell lines and cancer types

Technology Routes

Ubuntu 16.4.4

APACHE HTTP SERVER PROJECT — Apache 2.4.18

MySQL — Mysql 5.7.24

Python 3.5.2

Visualization Results

1. Pharmacological subtype tree

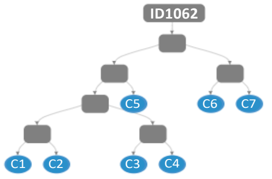

Drug Tree DataBase

Home | Search | Browser | Downloads | Help

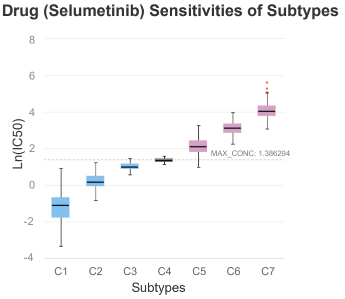

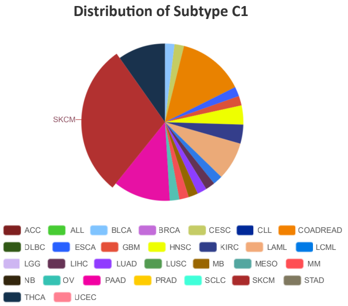

2. Expression and mutation/fusion pharmacological subtype

Show gene alteration connected with drug:

Show gene expression connected with drug:

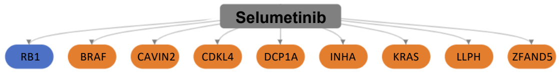

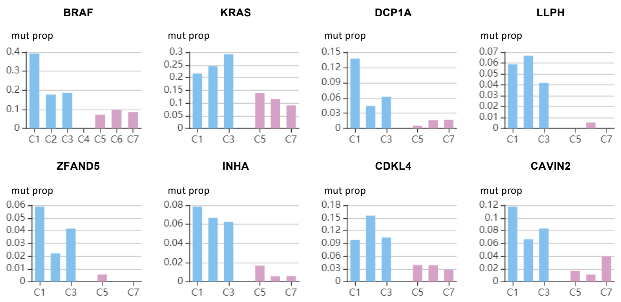

3. Cell similarity and drug similarity defined from the therapeutic concordance

e.g. drug: Quizartinib

Drug Id	Drug Name	Similarity	Synonyms	Target	Target Pathway
254	Quizartinib	1	AC220, AC 220, AC-220, Asp-2689	FLT3	RTK signaling
266	Zibotentan	0.221375421	ZD4054, ZD-4054	Endothelin-1 receptor (EDNRA)	Other
409	eEF2K Inhibitor, A-484954	0.217792491	NA	eEF2K	Other, kinases
1262	UNC1215	0.209496485	UNC-1215	L3MBTL3	Chromatin other
312	Tivozanib	0.207255414	AV-951, AV 951, KRN-951, KIL8951, ASP-4130	VEGFR1, VEGFR2, VEGFR3	RTK signaling
408	Sphingosine Kinase 1 Inhibitor II	0.206228505	PF-543	Sphingosine Kinase	Other, kinases
281	Alectinib	0.196800034	CH5424802, CH 542802, Alecensa	ALK	RTK signaling
