# Supplementary material for: Therapeutic Response-Based Reclassification of Multiple Tumor Subtypes Reveals Intrinsic Molecular Concordance of Therapy Across Histologically Disparate Cancers
Source: Front Cell Dev Biol. 2021 Nov 12;9:773101. doi: 10.3389/fcell.2021.773101 (PMC8632957; doi:10.3389/fcell.2021.773101)
Supplement: Supplementary file 7 [file Image4.PDF]

Figure S4

Gene	# of connection	Cancer gene
NQO1	44	No
CAPN2	43	No
PERP	42	No
PIP4K2C	38	No
CDC14B	33	No
BCAR1	30	No
CARD8	28	No
GNG12	26	No
UACA	26	No
WWTR1	26	Yes
BCL9L	25	Yes
ID1	25	No
IL13RA1	25	No
JUN	25	Yes
CBY1	24	No
NFATC3	23	No
RAC1	23	Yes
RRAS	23	No
CDC25A	22	No
E2F2	22	No
FANCD2	22	Yes
MCM5	22	No
MET	22	Yes
ORC1	22	No
PIK3CG	22	No
EGFR	21	Yes
FHL2	21	No
MCM6	21	No
MDM4	21	Yes
RASGRP2	21	No
