# Supplementary material for: Therapeutic Response-Based Reclassification of Multiple Tumor Subtypes Reveals Intrinsic Molecular Concordance of Therapy Across Histologically Disparate Cancers
Source: Front Cell Dev Biol. 2021 Nov 12;9:773101. doi: 10.3389/fcell.2021.773101 (PMC8632957; doi:10.3389/fcell.2021.773101)
Supplement: Supplementary file 8 [file Image2.PDF]

Figure S2

Gene	# of connection	Cancer gene
KCTD1_PCAT18	29	No
ABL1_BCR	28	Yes
BCR_ABL1	20	Yes
LTB	18	No
TNFRSF9	18	No
NFATC4	18	No
HLA-B	17	No
KPRP	15	No
KLHDC7A	15	No
KRAS	12	Yes
DERA	12	No
BRAF	12	Yes
TP53	12	Yes
RB1	11	Yes
NOTCH2	10	Yes
HLA-DMB	10	No
SUGP1	10	No
PCED1A	9	No
CTBP1_SPON2	9	No
MLLT3_KMT2A	8	Yes
SLFN12L	7	No
TET3	7	No
EZH2	7	Yes
MYC	7	Yes
UBC_NCOR2	7	Yes
JADE1	6	No
ULK4	6	No
MAFG	6	No
HYPM	6	No
RSPO4	6	No
